# Differentiation Between Abscesses and Unnecessary Intervention Fluid After Pancreas Surgery Using Dual-Energy Computed Tomography

**DOI:** 10.7759/cureus.62811

**Published:** 2024-06-21

**Authors:** Taro Tanaka, Kazuhiro Saito, Shuhei Shibukawa, Daisuke Yoshimaru, Hiroaki Osakabe, Yuichi Nagakawa, Yu Tajima

**Affiliations:** 1 Radiology, Tokyo Medical University, Tokyo, JPN; 2 Gastrointestinal and Pediatric Surgery, Tokyo Medical University, Tokyo, JPN

**Keywords:** virtual non-contrast image, ascites, abscess, pancreatic fistula, dual-energy computed tomography

## Abstract

Introduction: This study aimed to evaluate the potential of dual-energy computed tomography (CT) to distinguish postoperative ascites, pancreatic fistula, and abscesses.

Materials and method: Patients who underwent biliary and pancreatic surgery performed at our institution between June 2021 and February 2022 were included in the study. Postoperative body fluid samples were collected through a drain or percutaneous drainage. These samples were set in a phantom, and imaging data were obtained using dual-energy CT. Image analysis was performed to obtain CT values at each energy in virtual monoenergetic images (VMIs), effective atomic number, iodine map, and virtual non-contrast (VNC) images. VMIs were calculated from 80 and 140 kVp tube data at 10 kV each from 40-140 kV. Additionally, the effective atomic number, iodine map, and VNC images were reconstructed from the material decomposition process using water and iodine as the base material pair.

Results: In this study, 25 patients (eight with abscess and 17 with ascites) were included. No significant association was observed between the presence or absence of abscess and malignancy or surgical procedure. The intervention was performed in six of the eight patients with abscesses. In contrast, five of the 17 patients with postoperative ascites required intervention. A significant relationship was observed between the intervention and the presence of an abscess. Significant differences in C-reactive protein values and the incidence of fever were observed between the groups. Only VNC showed a significant difference between the groups.

Conclusions: VNC using dual-energy CT could differentiate abscesses from postoperative fluid.

## Introduction

The rate of operative complications of pancreatectomy is relatively high, reaching approximately 30% [1-3]. Among these operative complications, pancreatic fistula can develop a life-threatening complication. The International Study Group of Pancreatic Surgery (ISGPS) proposed that clinically relevant pancreatic fistulas require intervention [4,5]. Clinically relevant pancreatic fistulas are classified into grades B and C, accounting for approximately 15% of all cases [6]. A pancreatic fistula develops into peripancreatic collections, intraabdominal abscesses, delayed gastric emptying, and postoperative hemorrhage [7]. Among these operative complications, infectious fluid and hemorrhage are life-threatening complications. Clinical and biochemical parameters, such as infectious symptoms, elevated inflammation reaction, and high amylase-containing fluid from the drain, usually make us notice a pancreatic fistula. However, a biochemical leak formerly classified as a grade A pancreatic fistula has no clinical importance or need for intervention [5]. Therefore, physicians want to know rapidly whether intervention is needed for the postoperative fluid at the pancreatic bed. Post-operative hemorrhage can be diagnosed relatively easily due to the vital signs or typical radiological findings of extravasation of contrast media or high-density fluid collection. A local infection or intraabdominal abscess increases the morbidity rate of intraabdominal hemorrhage by 6-26% (8); furthermore, once single- or multi-organ dysfunction occurs, the mortality rate increases [5]. Therefore, correctly diagnosing abscesses or infectious fluid is essential. The diagnostic challenge for pancreatic fistulas on computed tomography (CT) has been explored [8]. The recent dual-energy CT (DECT) advance can distinguish between postoperative abscesses and other body fluids [9]. This ex vivo pilot study aimed to evaluate the ability of DECT to differentiate between abscesses and ascites, which were from a drain placed after pancreatic surgery.

## Materials and methods

The Ethics Committee of Tokyo Medical University approved this study (No. T2019-0264), and we obtained written informed consent from all patients.

Study subjects

**Figure 1:**
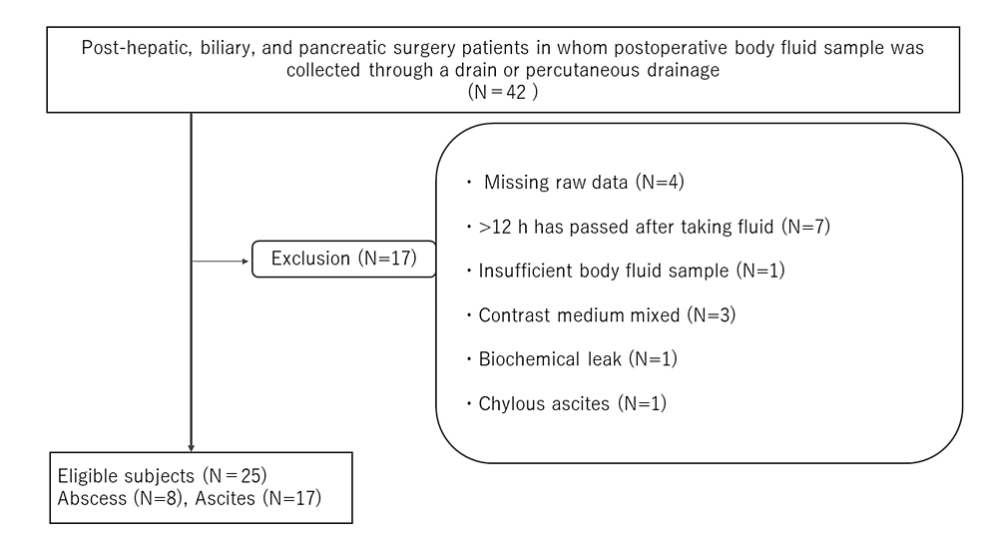
Patient selection This flowchart shows the inclusion and exclusion criteria for patient selection for this study.

Patients who underwent biliary and pancreatic surgery performed at our institution between June 2021 and February 2022 were included in the study. The inclusion criteria were as follows: the postoperative body fluid sample was collected through a drain or percutaneous drainage, and the patients were aged ≥20 years. The exclusion criteria were as follows: insufficient body fluid sample, body fluid sample was contaminated with contrast media, a blood biochemistry test was not performed on the same day in collecting body fluid, no bacteriological examination of the body fluid sample, no fresh body fluid sample (>12 h has passed after taking fluid), and biochemical leak [5]. The flowchart of the selection of the participants in this study is shown in Figure 1.

Body fluid sample definition

We divided the participants into two groups based on the bacteriological and biochemical tests. An abscess was defined as leukocyte phagocytosis or bacterial identification confirmed by Gram staining. Cases other than abscesses and biochemical leaks were classified as postoperative ascites [5].

CT scanning and postprocessing

All examinations were performed on a 256-detector DECT scanner (GE Healthcare, Milwaukee, WI, USA). Each fluid sample was scanned in a QA phantom (CIRS MECT 662). All CT scans were acquired using the following parameters: detector collimation, 256 × 0.625; default peak kilovoltage (kV1p) tube voltage, 80 and 140 kV; rotation time, 0.5 s; pitch, 0.508; and matrix size, 512 × 512.

Image analysis and measurement were performed using commercially available software (AW, version 4.7; GE, Healthcare). Virtual monoenergetic image (VMI) was calculated from 80 and 140 kVp tube data at 10 kV each from 40-140 kV. Additionally, effective atomic number (effective Z), iodine map, and virtual non-contrast (VNC) images were reconstructed from the material decomposition process using water and iodine as the base material pair. All CT images were reconstructed for a slice thickness of 5.0 mm, which is usually used in clinical practice.

Imaging analysis

**Figure 2:**
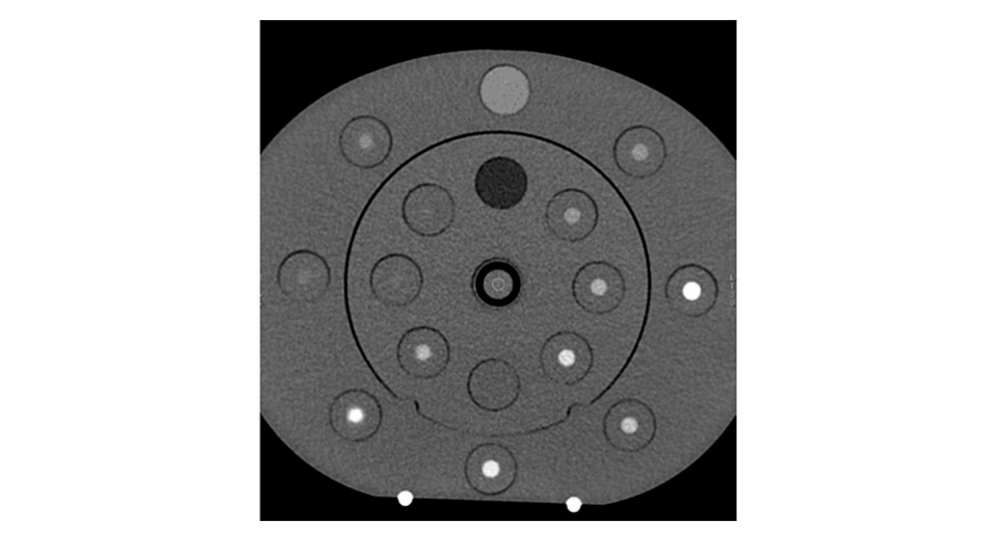
A phantom image in CT This computed tomography image shows a phantom. The center of the phantom is the specimen of body fluid. The artwork was created by one of the authors.

The same round region of interest (ROI), with a diameter of 50 pixels, was manually drawn on the VMI of the bottles filled with abscess and ascites using dedicated workstations (AW, version 4.7; GE, Healthcare) by one of the authors (10 years of experience as a certified diagnostic radiologist). Circular or oval ROIs were placed at the center of the bottle on each image, and 50 pixels were measured (Figure 2).

Statistical analysis

Differences in age, WBC, CRP, amylase level of the specimen, and fever were examined using the Mann-Whitney U test. For gender, comparisons between groups were made using Fisher's exact test. For each body fluid, the median and interquartile range (IQR) from 50 pixels were calculated. Shapiro-Wilk tests were used to confirm the normal distribution of these body fluid data. Due to the data not being normally distributed univariately, nonparametric tests were utilized for data analysis. The Mann-Whitney U test was employed to investigate differences between two body fluid groups in the analyses of the Hounsfield unit (HU), effective Z, iodine map, and VNC. A p-value of <0.05 was considered statistically significant. Effect sizes were calculated using the rank biserial correlation. HU, iodine map, and VNC were calculated at each kV with 95% confidence intervals (CIs). All statistical analyses were performed using R software (version 4.1.2, https://www.r-project.org).

## Results

Twenty-six patients satisfied both the inclusion and exclusion criteria, including eight patients with abscesses, 17 patients with ascites, and one patient with pancreatic fistula (corresponding to ISGPS grade 1). No hematoma or biliary fistula was suspected grossly or biochemically. The patient with pancreatic fistula was excluded from this study because of the difficulty in statistical analysis. Finally, 25 patients (eight patients with abscesses and 17 patients with ascites) were included in this study.

The patients’ underlying diseases and the type of surgical procedure are shown in Table 1. No significant associations were observed between the presence or absence of abscess and malignancy (p = 0.128) or surgical procedure (p = 0.651). The intervention was performed in six of the eight patients with abscesses (75%). In contrast, only five of the 17 patients with postoperative ascites (29%) required intervention. A significant relationship was observed between the intervention and the presence of abscess (p = 0.04). The causative bacteria of the abscess were as follows: *Pseudomonas aeruginosa* (n = 2), staphylococci (n = 2), *Streptococcus parasanguinis* (n = 1), *Escherichia coli* (n =1), *Enterococcus faecalis* (n = 1), and *Klebsiella oxytoca* (n = 1). The demographic and physical data in each patient group are shown in Table 2. Significant differences in C-reactive protein (CRP) values (p = 0.04) and the incidence of fever (p = 0.01) were observed between the two groups.

**Table 1:** The patients’ underlying diseases and the kind of surgical procedure IPMN: Intraductal papillary mucinous neoplasm, IR: Interventional radiology.

		Type of fluid collected by postoperative drain or IR	
		Abscess (n =8 )	Postoperative ascites (n = 17)
Disease	Bile duct cancer	2	2
	IPMN, IPMN-derived cancer	2	3
	Pancreatic cancer	0	10
	Neuroendocrine tumor	0	1
	Other malignancy	2	0
	Benign	2	1
Operation	Pancreatoduodenectomy	6	13
	Distal pancreatectomy	0	2
	Total pancreatectomy	0	1
	Extrahepatic bile duct resection	0	1
	Others	2	0

**Table 2:** The summary of demographics and laboratory results Data represent median (Interquartile Range: IQR). Asterisks indicate significance value (p < 0.05). C-reactive protein and fever show significant differences between abscesses and ascites. *Normal value is 3.3-8.6×103 /μL; **Normal value is 0.0 - 0.14 mg/dl; ***Normal value is less than 396 U/L.

Patient characteristics	Body fluid sample		Statistics for two sample groups
	Abscess	Ascites	P value
Age	75 [54.75 – 77.5]	71 [62 – 79]	0.887
Gender (male, female)	(7, 1)	(9, 8)	0.182
WBC*	10.05 [7.3 – 12.0]	8.7 [7.6 – 11.1]	0.793
CRP**	15.9 [13.3 – 18.7]	6.8 [3.5 – 12.2]	0.041*
Amylase level of specimen***	42 [33 – 150]	88 [20 – 127]	0.907
Fever	37.6 [37.1 – 38.1]	36.9 [36.7 – 37.3]	0.009*

Comparisons of each DECT parameter between the abscess and ascites groups

**Figure 3:**
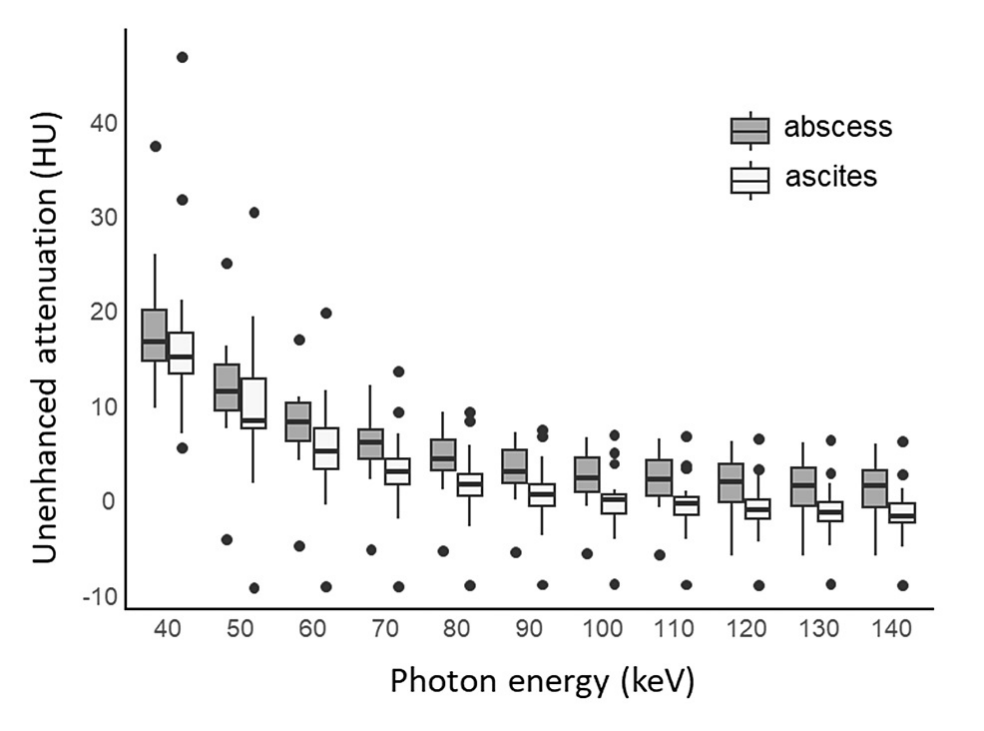
The spectral Hounsfield unit (HU) curve of the abscess and ascites This graph shows the spectral Hounsfield unit (HU) curve of the abscess (n = 8) and ascites (n = 17) groups. Boxes show the 25th–75th percentiles, and horizontal lines within the boxes represent the median values.

**Figure 4:**
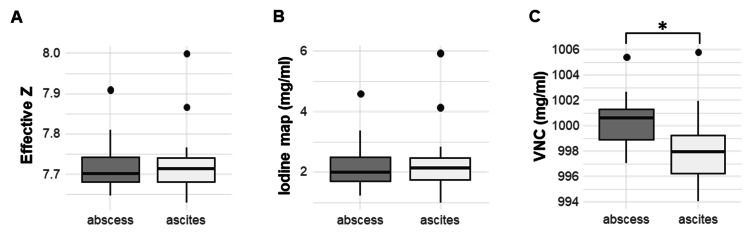
Box plots of effective Z, iodine map, and VNC images for the fluids of an abscess and ascites Box plots of (a) effective Z, (b) iodine map, and (c) virtual non-contrast (VNC) image for the fluids of an abscess (n = 8) and ascites (n = 17). Boxes show the 25th–75th percentiles, and horizontal lines within the boxes represent the median values. The asterisk indicates a statistically significant difference (p < 0.05).

All calculated parameters of the samples are shown in Table 3. The spectral HU curve is shown in Figure 3. CT values for each energy on a monochromatic image, effective Z, and iodine map showed no significant differences between the abscess and postoperative ascites groups (Table 3 and Figure 4). Only VNC showed a significant difference (p = 0.04) (Figure 4).

**Table 3:** Comparisons of each DECT parameter between abscess and ascites Data represent the median (IQR). Asterisks indicate significance value (p < 0.05). HU: Hounsefield units (unenhanced attenuation), Effective Z: Effective atomic number, DECT: Dual-energy CT.

Parameter		Body fluid sample		Statistics for two sample groups		
		Abscess: Median [IQR]	Ascites: Median [IQR]	P value	Effect size	95% CI
Spectral HU (kV)	40	17 (15, 20)	16 (14, 18)	0.50	0.18	-0.31 to 0.59
	50	12 (10, 14)	9 (8, 12)	0.28	0.28	-0.20 to 0.65
	60	8.7 (6.6, 10.7)	5.6 (3.6, 8.0)	0.19	0.34	-0.14 to 0.69
	70	6.5 (4.7, 7.9)	3.4 (2.1, 4.8)	0.19	0.34	-0.14 to 0.69
	80	4.8 (3.5, 6.8)	2.1 (0.9, 3.1)	0.12	0.40	-0.09 to 0.72
	90	3.4 (2.2, 5.7)	1.0 (-0.3, 2.1)	0.11	0.41	-0.14 to 0.73
	100	2.7 (1.3, 5.0)	0.5 (-0.9, 1.0)	0.10	0.43	-0.07 to 0.74
	110	2.6 (0.9, 4.6)	0.1 (-1.1, 0.8)	0.10	0.43	-0.06 to 0.74
	120	2.3 (0.2, 4.2)	-0.6 (-1.5, 0.4)	0.09	0.44	-0.04 to 0.75
	130	2.0 (-0.2, 3.8)	-0.9 (-1.8, 0.1)	0.10	0.43	-0.04 to 0.74
	140	1.9 (-0.4, 3.5)	-1.2 (-2.0, 0.1)	0.09	0.44	-0.02 to 0.75
Effective Z		7.70 (7.68, 7.74)	7.71 (7.68, 7.74)	0.88	-0.04	-0.49 to 0.42
Iodine map (mg/cm^3^)		2.00 (1.70, 2.48)	2.13 (1.74, 2.46)	0.88	-0.04	-0.49 to 0.42
Virtual non-contrast (mg/cm^3^)		1000.60 (998.91, 1001.29)	997.94 (996.22, 999.22)	0.037*	0.53	0.09 to 0.79

## Discussion

This study suggests that DECT can distinguish between an abscess and ascites. In this study, we attempted a VNC analysis instead of some ∆HU analysis or a simple spectral curve analysis that has been reported [10,11] because this study’s previous investigations were ineffective. VNC is the material decomposition image reconstructed using water and iodine as the base material pairs. Although abscesses and ascites usually do not contain iodine, the VNC value showed significantly higher abscesses than ascites. The spectral HU curve shows no significant differences between abscesses and ascites, although the HU in abscesses seems higher than in ascites. Furthermore, both values on the iodine map were not significantly different, although the average value of abscess was smaller than that of ascites. These findings indicate that the abscess may have a higher material density than the ascites. These results may be one of the reasons that the VNC value revealed the differences between the two entities. Additionally, Szczykutowicz TP has mentioned that this DECT phenomenon was a mathematical property: namely, the decomposition of any voxel not having one of the materials in the basis pair will result in the voxel appearing in images constructed with maps from either material [12].

In the neuroradiological field, non-contrast three-material decomposition images produced by VNC images can detect acute-phase stroke that cannot be seen by conventional non-contrast CT [13,14]. They assumed that VNC images highlighted parenchymal water, making it more evident than conventional non-contrast images. Postoperative ascites and abscesses are also fluid collections and have similar density. Abscesses contain necrotic tissue, bacteria, and blood; therefore, to some extent, the density may be high. In contrast, postoperative ascites are a collection of blood components that leak from blood vessels during inflammation and accumulate in tissues and on the surface of mucous membranes and contain leukocytes, inflammatory mediators, proteolytic enzymes, and cell growth factors, among others. The components of postoperative ascites and abscesses change depending on the type of wound, the healing process, and the presence or absence of infection, among others, and postoperative ascites may change into abscesses. Therefore, differentiating between the two entities must be difficult. We believe that VNC may contribute to the difference between the two entities.

The differences in HU between abscesses and ascites tended to be more prominent with higher keV on the monochromatic image. One of the reasons was that the structural components of abscesses and ascites were lower atomic number materials. The suppression of the photoelectric effect may partly affect it. Another reason could be decreased noise at higher keV. The contrast increased after the administration of contrast media at lower keV, which is well known; however, the signal-to-noise ratio increased. Noguchi et al. have reported that non-contrast DECT using higher keV more clearly showed acute stroke than non-contrast DECT using lower keV, and they mentioned that noise reduction was one of the reasons [14].

There are some limitations to this study. First, this study was a single-center study with limited cases. However, this analysis is the first experience and has valuable results. Second, this study is ex vivo, and clinical efficacy was not evaluated. Further study is warranted to confirm its clinical importance.

## Conclusions

In conclusion, this ex vivo pilot study demonstrated the potential of DECT to differentiate fluid retention between abscesses and postoperative ascites. We tried image analysis of CT values at each energy in virtual monoenergetic images, effective atomic number, iodine map, and virtual non-contrast (VNC) images, and only VNC showed a significant difference. VNC may be an effective tool for differentiating abscesses from postoperative ascites.

## References

[1] Greenblatt DY, Kelly KJ, Rajamanickam V (2011). Preoperative factors predict perioperative morbidity and mortality after pancreaticoduodenectomy. Ann Surg Oncol.

[2] Kneuertz PJ, Pitt HA, Bilimoria KY, Smiley JP, Cohen ME, Ko CY, Pawlik TM (2012). Risk of morbidity and mortality following hepato-pancreato-biliary surgery. J Gastrointest Surg.

[3] Vin Y, Sima CS, Getrajdman GI, Brown KT, Covey A, Brennan MF, Allen PJ (2008). Management and outcomes of postpancreatectomy fistula, leak, and abscess: results of 908 patients resected at a single institution between 2000 and 2005. J Am Coll Surg.

[4] Bassi C, Dervenis C, Butturini G (2005). Postoperative pancreatic fistula: an international study group (ISGPF) definition. Surgery.

[5] Bassi C, Marchegiani G, Dervenis C (2017). The 2016 update of the International Study Group (ISGPS) definition and grading of postoperative pancreatic fistula: 11 years after. Surgery.

[6] McMillan MT, Soi S, Asbun HJ (2016). Risk-adjusted outcomes of clinically relevant pancreatic fistula following pancreatoduodenectomy: a model for performance evaluation. Ann Surg.

[7] Butturini G, Daskalaki D, Molinari E, Scopelliti F, Casarotto A, Bassi C (2008). Pancreatic fistula: definition and current problems. J Hepatobiliary Pancreat Surg.

[8] Lee HJ, Kim JW, Hur YH (2019). Multidetector CT findings differ between surgical grades of pancreatic fistula after pancreaticoduodenectomy. Eur Radiol.

[9] Mahnken AH, Stanzel S, Heismann B (2009). Spectral rhoZ-projection method for characterization of body fluids in computed tomography: ex vivo experiments. Acad Radiol.

[10] Nagayama Y, Inoue T, Oda S (2021). Unenhanced dual-layer spectral-detector CT for characterizing indeterminate adrenal lesions. Radiology.

[11] Kim JE, Kim HO, Bae K, Cho JM, Choi HC, Choi DS (2017). Differentiation of small intrahepatic mass-forming cholangiocarcinoma from small liver abscess by dual source dual-energy CT quantitative parameters. Eur J Radiol.

[12] Szczykutowicz TP (2017). Hallway conversations in physics (why do i see iodine signal coming from bones on dual-energy CT images?). AJR Am J Roentgenol.

[13] Wolman DN, van Ommen F, Tong E (2021). Non-contrast dual-energy CT virtual ischemia maps accurately estimate ischemic core size in large-vessel occlusive stroke. Sci Rep.

[14] Noguchi K, Itoh T, Naruto N, Takashima S, Tanaka K, Kuroda S (2017). A novel imaging technique (X-Map) to identify acute ischemic lesions using noncontrast dual-energy computed tomography. J Stroke Cerebrovasc Dis.

